# A New Phenomenon: Sub-T_*g*_, Solid-State, Plasticity-Induced Bonding in Polymers

**DOI:** 10.1038/srep46405

**Published:** 2017-04-20

**Authors:** Nikhil Padhye, David M. Parks, Bernhardt L. Trout, Alexander H. Slocum

**Affiliations:** 1 Massachusetts Institute of Technology, Department of Mechanical Engineering, Cambridge, 02139, USA; 2 Massachusetts Institute of Technology, Department of Chemical Engineering, Cambridge, 02139, USA

## Abstract

Polymer self-adhesion due to the interdiffusion of macromolecules has been an active area of research for several decades. Here, we report a new phenomenon of sub-T_*g*_, solid-state, plasticity-induced bonding; where amorphous polymeric films were bonded together in a period of time on the order of a second in the solid-state at ambient temperatures, up to 60 K below their glass transition temperature (T_*g*_), by subjecting them to active plastic deformation. Despite the glassy regime, the bulk plastic deformation triggered the requisite molecular mobility of the polymer chains, causing interpenetration across the interfaces held in contact. Quantitative levels of adhesion and the morphologies of the fractured interfaces validated the sub-T_*g*_, plasticity-induced, molecular mobilization causing bonding. No-bonding outcomes (i) during the uniaxial compressive straining of films (a near-hydrostatic setting which strongly limits plastic flow) and (ii) between an ‘elastic’ and a ‘plastic’ film further established the explicit role of plastic deformation in this newly reported sub-T_*g*_ solid-state bonding.

If two pieces of a glassy polymer are brought into molecular proximity at temperatures well below their glass transition temperature (T_*g*_), negligible adhesion due to interdiffusion of macromolecules will be noted. Because polymer chains are kinetically trapped well below the T_*g*_^1^,^2^,^3^,^4^, the time scales for relaxations in the glassy state are extremely large^5^,^6^,^7^. Therefore, the system is essentially frozen with respect to any cooperative segmental motions (*α*-like relaxation)^8^ that would cause interdiffusion. The glass transition temperature itself is typically characterized by viscosity and diffusivity values of 10^13^ Poise and 10^−24^ m^2^/s, respectively^9^. Assuming a viscosity of 10^13^ Poise at the glass transition temperature^10^, self-diffusion coefficients of forty polymers (at their respective glass-transition temperatures) were estimated to be approximately 10^−25^ m^2^/s. Similarly, several other examples of extremely slow kinetics in glass forming liquids near the T_*g*_ (marked by very small diffusion coefficients) are reported in the literature^11^,^12^,^13^.

However, if the two pieces are brought into contact at a temperature above the glass transition temperature, along with the application of moderate contact pressure, polymer chains from the two sides interdiffuse on experimental timescales^14^,^15^,^16^,^17^,^18^,^19^,^20^,^21^,^22^,^23^. As a result of this interdiffusion, there is an optical disappearance of cracks and the development of strong bonds between the two surfaces over time. The strength of the developing interface is a function of temperature, time of healing and contact pressure, and the healing process continues until the interface acquires the bulk properties. Typically, for times smaller than the bulk reptation time, the interface toughness (G_*c*_) and shear strength (*σ*_*s*_) show a monotonic time-dependent growth as G_*c*_ ~ *t*^1/2^ and *σ*_*s*_ ~ *t*^1/4^ 22,24,25,26. The temperature strongly dictates the molecular mobility, with the self-diffusion coefficient of polymer melts usually ranging between 10^−10^ and 10^−20^ m^2^/s (see Supplementary Table 3). Moderate contact pressures (ranging from 0.1 MPa to 0.8 MPa) have been reported to be essential for facilitating the intimate contact between the interfaces that allows interdiffusion. The chemical structure, the molecular weight and polydispersity of the polymer, the geometry of the joint, and the method of testing are critical factors affecting the measured strength or toughness of the interface.

In the past two decades, there have been reports of evolving polymer adhesion due to interdiffusion at temperatures somewhat below the bulk T_*g*_, with relatively long healing times of order several minutes^27^,^28^, hours^29^,^30^, and even up to a day^31^. Interpretations of such studies have suggested that bonding of a glassy polymer via molecular interdiffusion, even at temperatures below the bulk T_*g*_, is possible over such time scales if a near-surface layer of the polymer remains in a rubbery state. Evidence of enhanced molecular dynamics characteristic of rubbery-like behavior, taking place within a thin layer at the free surface of an otherwise glassy polymer, has been obtained from both experiments^32^,^33^ and computer simulations^34^. Within near-surface layers, the component of mean macromolecular orientation normal to the free surface is suppressed. The resultant effects of entropic and enthalpic factors can lead to segregation or repulsion of chain ends at the free surface^35^. The segregation of chain ends at the free surface can contribute to the depression of the glass transition temperature at the surface^35^,^36^,^37^,^38^. However, such effects decay within distances from the free surface comparable to the bulk radius of gyration of the polymer.

Although the motion of macromolecules in a glassy state is effectively frozen on short time scales, stress-induced molecular mobility of glasses has been studied since the work of Eyring^39^. Argon and co-workers^40^ demonstrated that the case II sorption rates of low molecular weight diluent species into a plastically-deforming glassy poly(ether-imide) were dramatically enhanced, and were comparable with the sorption rates into the polymer at T_*g*_, and that plastically-deforming glassy polymers exhibit a mechanically-dilated dynamical state bearing strong similarities to the molecular-level conformational rearrangements taking place at T_*g*_ in the absence of active deformation. A related study^41^ also reported an increase in the case II front velocity (of approximately 6.5 times) when an out-of-surface tensile stress was applied. Lee *et al*.^42^ showed that uniaxial deformation of PMMA 19 K below its T_*g*_ exhibited an increased molecular mobility by up to 1000 times. Loo *et al*.^43^ used NMR to probe deuterated semi-crystalline Nylon 6 and reported enhanced conformational dynamics in the amorphous regions of Nylon when deformation was carried out near T_*g*_. Molecular dynamics simulations^44^ also revealed increased torsional transition rates and thus enhanced molecular mobility during active deformation of a glass. The plastic deformation of glassy polymers is understood in terms of localized step-like shear cooperative displacements of lengthy chain segments, and the unit plastic rearrangements are known as shear transformations^45^. According to molecular dynamics simulations^46^, slippage of chains is the underlying feature of a shear transformation (for a detailed discussion, see Supplementary Section 3.2). Here, we report that active plastic deformation of glassy polymeric films held in intimate contact can trigger requisite molecular-level rearrangement sufficient to cause interpenetration of polymer chains across the interface, which leads to bonding. Figure 1 compares and contrasts cases of polymer self-adhesion through interdiffusion with the plasticity-induced bonding mechanism proposed herein.

## Results

**Figure 2:**
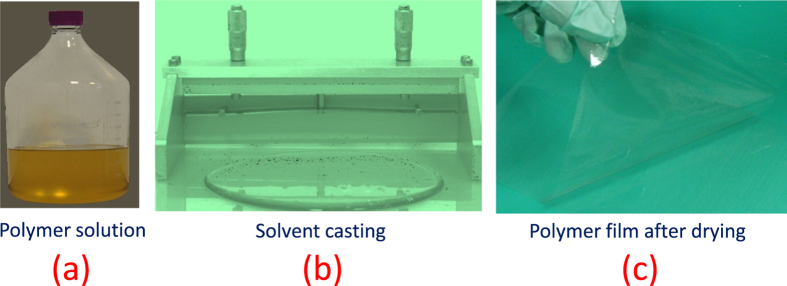
Steps involved in the preparation of polymer films through solvent casting: (**a**) homogeneous solution of polymer and plasticizer in ethanol and water, (**b**) spreading of the solution on a glass surface via a knife, and (**c**) evaporation of solvents and formation of a glassy film after drying.

**Figure 3:**
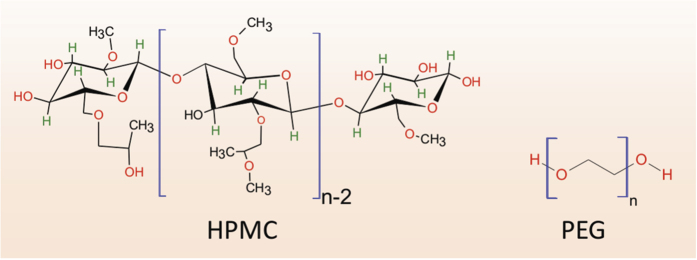
Molecular structures of hydroxypropyl methylcellulose (HPMC) and polyethylene glycol (PEG).

**Figure 4:**
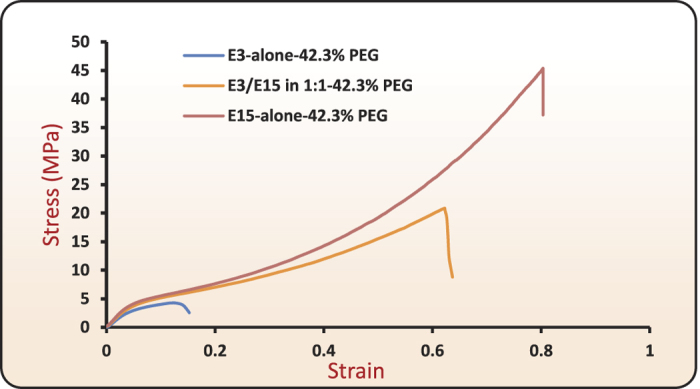
Ambient-temperature tensile true stress- true strain curves for three film formulations: E3-alone-42.3% PEG, E3/E15 in 1:1–42.3% PEG and E15-alone-42.3% PEG. The nominal strain rate for tensile testing was 0.0025 sec^−1^.

Figure 2 illustrates the preparation of polymeric films by solvent casting using a base polymer (hydroxypropyl methylcellulose) and a plasticizer (polyethylene glycol, PEG-400). The base polymer HPMC was available under the trade name METHOCEL in E3 and E15 grades. The molecular structures of the polymer and plasticizer are shown in Fig. 3. Films of varying composition were prepared and assigned unique names characterizing base polymer and weight percent (wt.%) of the plasticizer in the film with respect to the base polymer (see Methods and Supplementary Section 1). Films made from E3-alone-42.3% PEG, E3/E15 in 1:1–42.3% PEG and E15-alone-42.3% PEG exhibited T_*g*_-values in the range of 72–78 °C. (See Methods and Supplementary Section 2.2). Their ambient-temperature tensile true stress-strain curves are shown in Fig. 4. All three films exhibited ductility, represented by their ability to undergo plastic flow.

**Figure 5:**
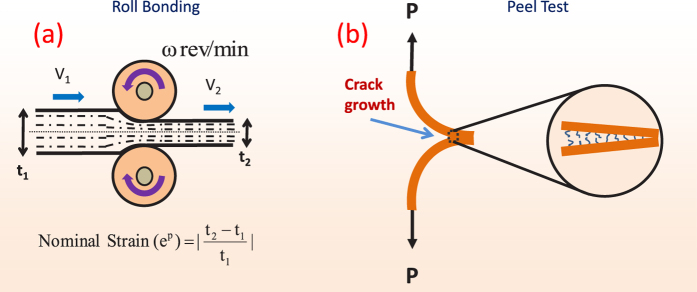
(**a**) Roll-bonding was achieved by passing a stack of film layers with a total initial thickness t_1_ between compression rollers to yield a final-thickness t_2_. (**b**) The peel-test was carried out on a roll-bonded sample, forcing delamination at the middle interface.

**Figure 6:**
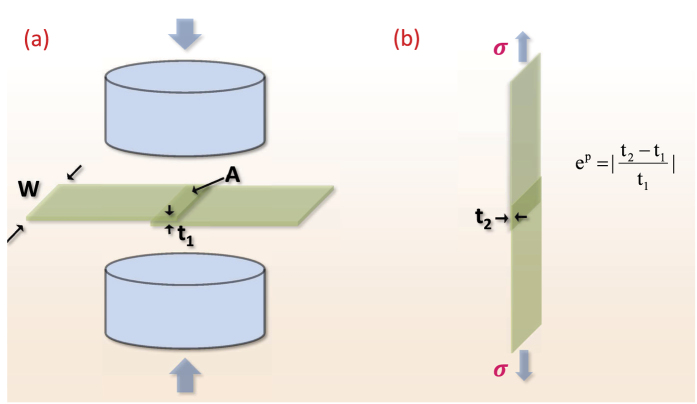
Lap specimens were prepared between two film layers. (**a**) Application of compression loads on the overlapping area (*A*) to cause plastic deformation. Initial thickness (*t*_1_) is reduced to (*t*_2_). (**b**) Lap shear-strength measurements were performed in a tensile mode.

Bonding experiments were carried out at ambient conditions (18° ± 2 °C). (i) Stacks of six film layers, each of thickness ~100 *μ*m, were fed through a roll-bonding machine to achieve active plastic deformation at ambient temperatures over time intervals on the order of a second (see Supplementary Table 4 for estimates of the active deformation times due to rolling). Symmetric peel tests were performed to measure the mode I fracture toughness (G_*c*_ [J/m^2^ ]), Fig. 5, and (ii) lap specimens were prepared to measure the shear-strength (*σ*_*s*_ [MPa]), Fig. 6. (See Methods and Supplementary Section 4 for details on roll-bonding, peel testing and lap shear strength testing). G_*c*_ represents the work done per unit area for debonding the interface during a peel test. *σ*_*s*_ indicates the maximum nominal shear stress sustained by the bonded interface before failure. The effective thickness reduction was used as a measure of plastic strain during bonding in all of the cases.

**Figure 7:**
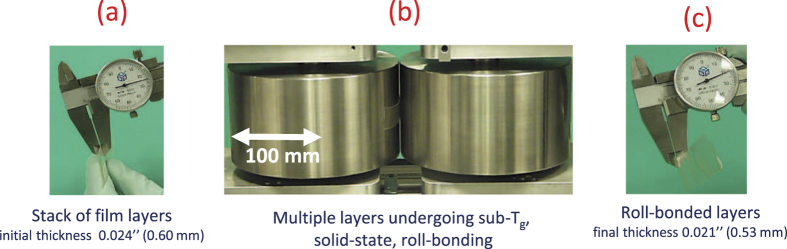
Illustration of sub-T_*g*_, solid-state, plasticity-induced roll-bonding of E3/E15 in 1:1–42.3% PEG films nearly 60 K below T_*g*_. For this case, the nominal thickness strain is e_*p*_ = |*t*_2_ − *t*_1_|/*t*_1_ = 11.7%.

Figure 7 illustrates the consolidation of several layers of the film (E3/E15 in 1:1–42.3% PEG) comprising an initial thickness of t_1_ = 0.60 mm, which undergoes roll-bonding through active plastic deformation, emerging from the process with an integral final thickness reduced to t_2_ = 0.533 mm (see Supplementary video S1).

**Figure 8:**
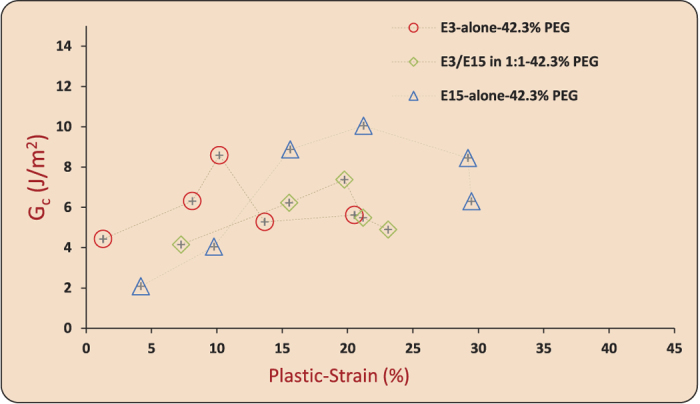
Fracture toughness (G_*c*_ [J/m^2^]) versus plastic strain plots for E3/E15 in 1:1–42.3%PEG, E3-alone-42.3%PEG and E15-alone-42.3%PEG. The G_*c*_ values are based on the mean steady-state force during peeling and the error bars in G_*c*_ correspond to fluctuations during steady-state peeling. Plastic strain is calculated based on 10 mean thickness measurements before and after bonding, and the error bars in plastic strain are derived from these measurements. The error bars in G_*c*_ and plastic strain represent uncertainty of one standard deviation.

**Figure 9:**
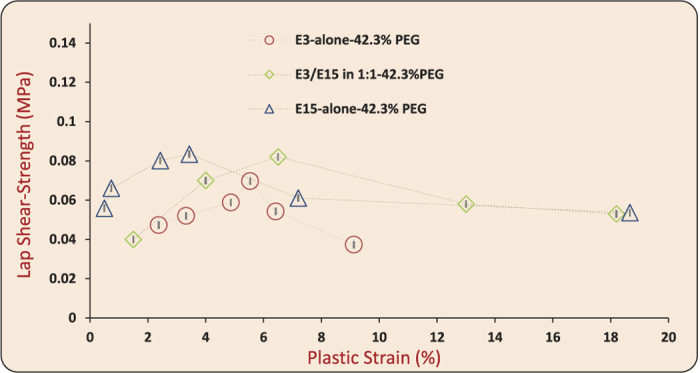
Lap shear-strength (*σ*_*s*_ [MPa]) versus plastic strain plots for E3/E15 in 1:1–42.3%PEG, E3-alone-42.3%PEG and E15-alone-42.3%PEG. Plastic strain is calculated based on mean thicknesses before and after bonding (10 measurements), and the error bars in plastic strain are derived from these measurements. The error bars on plastic strain represent uncertainty of one standard deviation.

Figure 8 shows G_*c*_ results for the three films. G_*c*_ correlates with the imposed plastic strain in a non-monotonic fashion, first increasing and then decreasing. The adhesion between two interfaces held together by van der Waals forces, hydrogen bonds, or chemical bonds can only give G_*c*_ values in the range of 0.05 J/m^2^. 0.1 J/m^2^ and 1.0 J/m^2^, respectively^47^. The surface energy of glassy polymers itself is quite small^48^ (on the order of 0.08 J/m^2^); therefore, negligible adhesion is noted when two such surfaces are brought into mere molecular proximity. However, glassy polymers can exhibit higher fracture toughness owing to the irreversible deformation of the macromolecules. Thus, the quantitative levels of interface toughness G_*c*_ obtained here, with a maximum value nearly 10 J/m^2^, can be attributed to the irreversible processes of chain pull-outs, disentanglement and/or scissions during debonding, which could only happen if plasticity-induced molecular mobilization and chain-interpenetration had led to bonding. It is worth emphasizing that studies on polymer adhesion leading to G_*c*_ values up to 1.2 J/m^2^ and 2.0 J/m^2^, respectively^31^,^49^, have attributed such levels of fracture toughnesses due to chain interdiffusion and irreversible chain pull-out mechanisms during debonding. The levels of fracture toughnesses reported in our study are comparably larger than those reported in these studies, and therefore affirm that in our case bonding occured via chain interpenetration and debonding involves irreversible chain pull-out processes. Other mechanisms of adhesion such as acid-base interactions, capillary effects, electrostatic forces and/or any other conceivable mechanism do not apply in the current context (for a detailed discussion on the types of forces giving rise to adhesion, see ref. 50). The lap shear strength (*σ*_*s*_) data, shown in Fig. 9, also exhibits a similar non-monotonic correlation with the bonding plastic strain. Quantitative levels of the *σ*_*s*_ values reported here compare with certain experimental results reported in the literature^29^, in which adhesion due to interdiffusion of chains up to 50 K below the bulk T_*g*_ over long times (on the order of several minutes) was reported. The reported levels of bulk plastic strains necessary for bonding also rule out any major role of mechanical interlocking of asperities to cause adhesion; at the levels of plastic strains reported here, surface asperities would necessarily flatten out. Surface characterization of the films through AFM, before bonding, revealed nano-scale roughness (R_*a*_) on the order of 6.91–22.7 nm (see Supplementary Section 2.5). By contrast, increasing levels of plastic strain lead to asymptotically-increasing contact areas, and if factors other than chain interpenetration were responsible for bonding, we would expect a monotonic increase in G_*c*_ or *σ*_*s*_. The decrease of G_*c*_ or *σ*_*s*_ at high levels of plastic strain could plausibly be explained on the basis of deformation-induced anisotropy in the near-interface chain orientation distribution. We suggest that increasing plastic strain ultimately causes increasing chain orientation parallel to the rolling direction (maximum principal stretch direction) that leads to less effective chain interpenetration across the interface, serving to reduce the degree of bonding at higher strains. We also studied the effect of strain-rate on roll-bonding of laminates and found that fracture toughness again correlated primarily with plastic strain, showing negligible strain-rate sensitivity (see Supplementary Figure 20 and discussion in Section 5).

**Figure 10:**
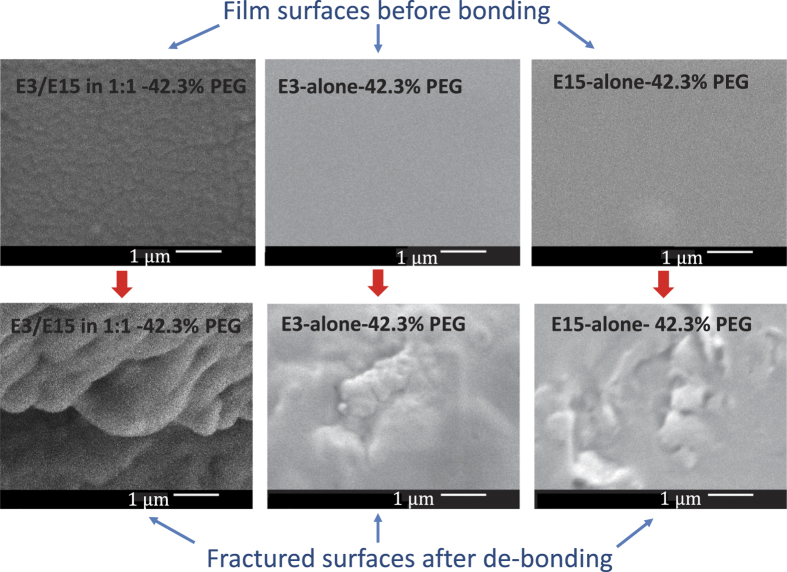
SEM images of E3/E15 in 1:1–42.3% PEG, E3-alone-42.3% PEG, and E15-alone-42.3% PEG, films before bonding and after debonding. The nominal plastic strains during roll-bonding for E3/E15 in 1:1–42.3% PEG, E3-alone-42.3% PEG, and E15-alone-42.3% PEG, films were 15.53%, 8.12%, and 10.18%, respectively.

Figure 10 shows a comparison of representative surface morphology before bonding and after the fracture. The debonded fracture surfaces indicate local sites of chain scissions or pull-outs due to fracture. Such features are similar to those reported upon fracture of polymers welded through interdiffusion^22^,^28^,^51^.

**Figure 11:**
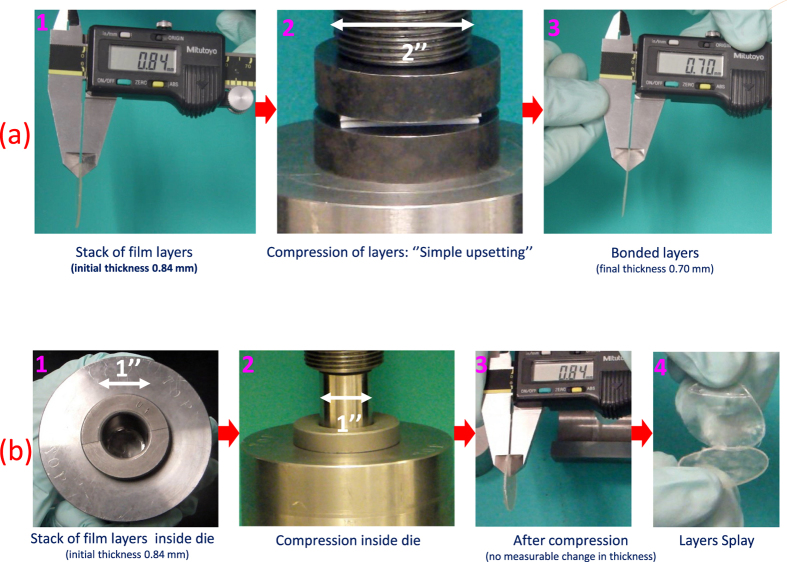
Compression of stacks of films (**a**) without any die containment to permit macroscopic plastic flow and bonding, (**b**) in a ‘uniaxial strain die’ that is capable of limiting the plastic flow, and consequently no bonding takes place. In both cases peak nominal compressive stresses (78.98 MPa) was kept same.

To explicitly demonstrate the role of bulk plastic deformation in bonding, we designed a ‘uniaxial die’ setup, which was capable of imposing uniaxial strain and strongly limiting the magnitude of macroscopic plastic flow. Figure 11 shows a comparison in which a stack of films (E3/E15 in 1:1–42.3% PEG) was compressed (i) without any constraints and (ii) with the ‘uniaxial die’ constraint. In both cases, the stack of films were subjected to same level of peak nominal compressive stress of 78.98 MPa for a short time interval on the order of a second. (See Supplementary Section 4.5 for deformation analyses and details). In the first case (simple upsetting), the stack underwent macroscopic compressive plastic flow, and the layers bonded to form an integral structure (see Supplementary video S2-a), whereas in the case of the ‘uniaxial die’ constraint, no permanent thickness change or plastic strain was observed, and the layers readily splayed apart after removal from the die (see Supplementary video S2-b part I, part II and part III).

**Figure 12:**
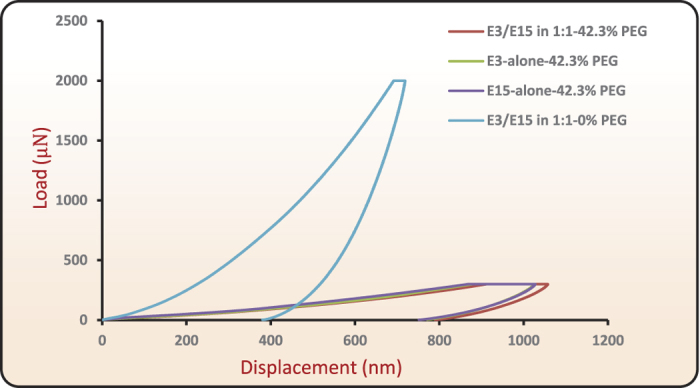
Illustration on load versus displacement curves in nanoindentation for E3/E15 in 1:1–0% PEG, E3/E15 in 1:1–42.3% PEG, E3-alone-42.3% PEG and E15-alone-42.3% PEG films. Indentation experiments were carried out in load controlled mode with chosen peak loads up to 300 *μ*N and 2000 *μ*N for films with 42.3% PEG and 0% PEG, respectively.

**Figure 13:**
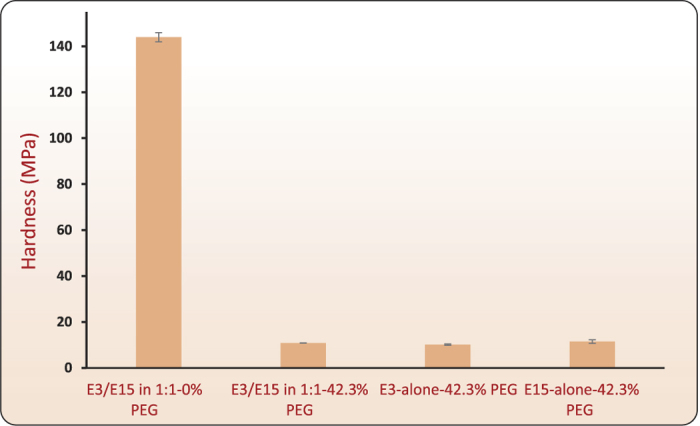
Hardness measurements for E3/E15 in 1:1–0% PEG, E3/E15 in 1:1–42.3% PEG, E3-alone-42.3% PEG and E15-alone-42.3% PEG films. Nanoindentation experiments were conducted on film surfaces of size 1 × 1 mm^2^ and the mean hardness over 51 measurements is shown. The error bars represent uncertainty of one standard deviation.

In another experiment, we attempted to roll-bond E3/E15 in a 1:1–0% PEG film with E3/E15 in a 1:1–42.3% PEG film (see Supplementary video S3) and a no-bonding outcome was noted. Films with 0% PEG have high flow strengths, and exhibit negligible plastic flow (see Supplementary Fig. 1(a)) when tested in tension. When attempting to roll-bond high-strength laminae to those of much lower flows strength, essentially all plastic deformation localizes within the lower-strength material. Because the higher-strength material remained glassy and non-deforming during rolling, it was therefore incapable of either contributing or incorporating any plasticity-mobilized chain segments across the interface, again leading to non-bonding results. Nanoindentation experiments were performed on E3/E15 in 1:1–0% PEG, E3/E15 in 1:1–42.3% PEG, E3-alone-42.3% PEG and E15-alone-42.3% PEG films. The indentation experiments were carried out in a force controlled mode with a maximum force of 2000 *μ*N and 300 *μ*N for 0% PEG and 42.3% PEG films, respectively. A larger load for the 0% PEG film was chosen in order to activate sufficient plastic indentation so that its hardness could be measured. Berkovich indenter with a root radius of 150 nm was used. The load versus displacement curves for all the films are shown in the Fig. 12. The film with 0% PEG shows a relatively large indentation force and large elastic recovery, whereas films with 42.3% PEG films show little elastic recovery and large residual indentation depth. Based on these relative behaviors, the 0% PEG film can be called an ‘elastic’ film and the 42.3% PEG film as a ‘plastic’ film. Using Oliver-Pharr method we estimated the hardness from the nano-indentation tests. The hardness values for E3/E15 in 1:1–0% PEG, E3/E15 in 1:1–42.3% PEG, E3-alone-42.3% PEG and E15-alone-42.3% PEG films were 144.0 ± 0.39 MPa, 10.83 ± 0.03 MPa, 10.151 ± 0.15 MPa, and 11.48 ± 0.39 MPa, respectively. The bar graph in Fig. 13 compares the hardness values of these films. This also confirmed, that at a given load, films with 0% PEG are “hard” and unlikely to demonstrate plasticity-induced molecular mobilization for bonding, whereas 42.3% PEG films can exhibit substantial plastic flow.

## Discussion

When the temperature of a glass-forming liquid is lowered and the glass transition temperature is approached from above, kinetics of a glass-forming system shows a drastic slow-down, and time scales for relaxations increase by orders of magnitude to allow any appreciable diffusion in experimental timescales. The classic Bueche-Cashin-Debye equation^52^,^53^, which relates diffusivity and viscosity, is given as:


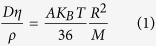


In the above equation, *A* is the Avogadro constant, *K*_*B*_ is the Boltzmann constant, *T* is the absolute temperature, *R*^2^ is the mean-square end-to-end distance of a single polymer chain, and *M* is the molecular weight. If we estimate *D* for our polymer at the glass-transition temperature by considering *η* = 10^13^ Poise, *ρ* = 1180 Kg/m^3^, R^2^ = 6 × 7.4^2^ nm^2^ (using R_*g*_ of E3, and R^2^ = 6 × 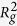) and *M* = 20,300 g/mol (See Supplementary Section 2.3 for molecular properties), and *T* = *T*_*g*_ = 352 K, the estimated value of D is 1.12 × 10^−24^ m^2^/s. This is a remarkable estimate in terms of the order of magnitude and compares well with the reported^10^ self-diffusivity of 10^−25^ m^2^/s. If we consider a scenario: in which a diffusion distance of x = 10 nm is to be achieved within a time interval of one second, then *D*(=*x*^2^/2*t*) must be greater than 0.5 × 10^−16^ m^2^/s. This is not possible in the solid-state, 60 K below the bulk-T_*g*_, and clarifies the distinction of polymer welding above T_*g*_ with respect to newly reported plasticity-induced molecular mobilization and bonding which occurred in a period of time on the order of a *second*.

Quantitative levels of bonding reported in this paper and their correlations with bulk plastic deformation and fracture surface morphology imply molecular mobilization and chain interpenetration across the interface as the mechanism of bonding. Furthermore, no-bonding outcomes in the ‘uniaxial die’ experiment and between an ‘elastic’ and a ‘plastic’ film emphasize that even effects associated with the presence of a rubbery-like layer of higher molecular mobility within a molecular thin layer near the surface, could not provide adhesion of the magnitude observed during contacts lasting on the order of a second at 50 C below T_*g*_. Both these experiments explicitly demonstrated that activating bulk plastic flow on both sides of the interface was an essential requirement for bonding. Bonding below the bulk T_*g*_ (without any bulk plastic deformation), as reported in the literature, requires substantially longer durations. Additionally, the existence of any enhanced relaxation of the polymer chains (or segments) in the surface layer would be severely restricted by any portions of the macromolecules extending into the glassy-bulk beneath; hence, long-range diffusion within a short time is not possible. Finally, although not considered in these prior reports, it is plausible that moderate contact pressures, applied over relatively long healing times at temperatures near T_*g*_, may have contributed to mechanically enhanced molecular mobility that contributed to bonding via mechanisms similar to those described here.

We emphasize that the kinetically trapped state of a molecular glass implies that any cooperative segmental relaxations or long range diffusive motions of chains are severely restricted; however, secondary relaxation processes (those corresponding to vibrations of side groups like *β, γ, δ*, etc. relaxations) may still be active. But, such weak secondary relaxation processes are incapable of giving any appreciable molecular interdiffusion and pronounced adhesion in a short-time (fraction of a second), when two interfaces are brought together in molecular proximity, unless enhanced mobility is triggered through plastic deformation.

Although rapid plastic deformation can cause a temperature rise, at relatively slow strain-rates the associated temperature rise is negligible. A fully adiabatic analysis revealed an upper bound temperature increase of only 3.6 °C (see Supplementary Section 3.3). The mechanically activated polymer mobility well below T_*g*_ is mechanistically quite different from molecular mobility at temperatures above T_*g*_, and the key differences can be summarized as follows: Diffusion primarily occurs due to high kinetic energy of the polymer chains (or segments), and available free-volume (or physical space) due to which chains (or segments) can sample new orientations effectively. The polymer melts (above T_*g*_) are spatially homogeneous and in a thermodynamic equilibrium state, whereas, plastic deformation and associated enhanced mobility in a glassy polymer is not at all an equilibrium concept. The root mean square displacement of center of mass of a polymer chain will increase monotonically with time during diffusion in a polymer melt, however, the mechanically assisted enhanced mobility in polymers only occurs during active plastic deformation and effectively ceases when plastic straining stops. The average kinetic energy of a polymer molecule is large in a polymer melt compared to that in the solid-state glass well below T_*g*_. Finally, the self-diffusion coefficient (D) of a polymer chain in its melt state shows a strong dependence on the molecular weight, D ~ M^−1^ or D ~ M^−2^ in accordance with the Rouse or the reptation model, respectively. However, all three blends of polymer considered here, E3-alone, E15-alone and E3/E15 in 1:1, were roll-bonded in time intervals on the order of a second, which, owing to the large differences in molecular weight among the blends, is in significant contrast from the mechanism of polymer adhesion due to interdiffusion. We speculate that shear transformation units of plastic deformation accompanied by local transient dilatations (volume changes) could facilitate opportunities for establishing entanglements across the interface, such that plasticity-induced bonding can take place in a period of time on the order of a second at temperatures many tens of degrees below bulk T_*g*_. Novel insights associated with the newly reported phenomena and proposed underlying mechanisms are expected to open new avenues for research and applications. Particularly, detailed mechanistic understanding of deformation induced polymer mobility causing bonding at the interface and their dependence on strain-rate, temperature etc. are worthwhile pursuits.

## Methods

### Film-Making

Hydroxypropyl methyl cellulose (HPMC), trade name METHOCEL, in grades E3 and E15 was obtained from Dow Chemical (Midland, Michigan, North America). PEG-400 was purchased from Sigma-Aldrich (Milwaukee, Wisconsin, North America). Appropriate amounts of E3, E15 and PEG were mixed in desired amounts with ethanol and water, and a homogeneous solution was obtained through mixing with an electric stirrer for 24 h. After completion of the blending process, the solution was carefully stored in glass bottles at rest for 12 h to eliminate air bubbles. Solvent casting was carried out using a casting knife applicator from Elcometer (Rochester Hills, Michigan, North America) on heat-resistant borosilicate glass. All of the steps were carried out in a chemical laboratory where ambient conditions of 18° ± 2 °C and R.H. 20 ± 5% were noted. The residual moisture content in the films after drying was measured using Karl Fischer titration.

### Bonding Experiments

Roll bonding was carried out on a machine capable of exerting the desired load levels to achieve active plastic deformation. A pair of 200 mm diameter rollers were driven at an angular speed of 0.5 rev/min, leading to an exit speed of 5.23 mm/s. Peel tests were carried out to measure mode I fracture toughness (see Supplementary video S4). Lap specimens were prepared using compression platens on an Instron mechanical tester. For both roll-bonded and lap specimens, for the sake of consistency, the adhesion measurements were carried out on the bonded interfaces between the top-top surfaces (exposed side during drying). Film layers were stacked accordingly. Top-bottom and bottom-bottom joining led to similar bonding results. The ‘uniaxial die’ and ‘upsetting’ experiments were carried out on the Instron. The roll-bonding machine and fixture for the peel test were designed and fabricated in Massachusetts Institute of Technology (Cambridge, North America) (see Supplementary Section 4).

### Characterization

The molecular weights of E3 and E15 were estimated from viscosity measurements. The amorphous nature of the films were verified by XRD. SEM and AFM were performed to analyze the surfaces. DMA was performed to determine the T_*g*_. Tensile stress-strain curve tests, fracture toughness through peel tests, and lap shear tests were carried out. Nanoindentation was carried out to measure the hardness. The specific heat capacity was measured using DSC.

X-ray diffraction was conducted using a PANalytical X’Pert PRO Theta/Theta powder X-ray diffraction system with a Cu tube and an X’Celerator high-speed detector. AFM images were obtained using a Dimension 3100 XY closed loop scanner (Nanoscope IV, VEECO) equipped with NanoMan software. Height and phase images were obtained in tapping mode in ambient air with silicon tips (VEECO). DMA was carried out on a TA Q800 instrument. Mechanical testing was performed on an Instron mechanical tester. Nanoindentation tests were carried out on a Triboindenter Hysitron instrument. Calorimetry was performed on a TA Q200 instrument. The viscosity was measured on an HR-3 Hybrid rheometer^54^.

## Additional Information

**How to cite this article**: Padhye, N. *et al*. A New Phenomenon: Sub-T_g_, Solid-State, Plasticity-Induced Bonding in Polymers. *Sci. Rep.* **7**, 46405; doi: 10.1038/srep46405 (2017).

**Publisher's note:** Springer Nature remains neutral with regard to jurisdictional claims in published maps and institutional affiliations.

## Supplementary Material

Supplementary Video S1

Supplementary Video S2a

Supplementary S2b part I

Supplementary S2b part II

Supplementary S2b part III

Supplementary Video S3

Supplementary Video S4

Supplementary Information

## Figures and Tables

**Figure 1:**
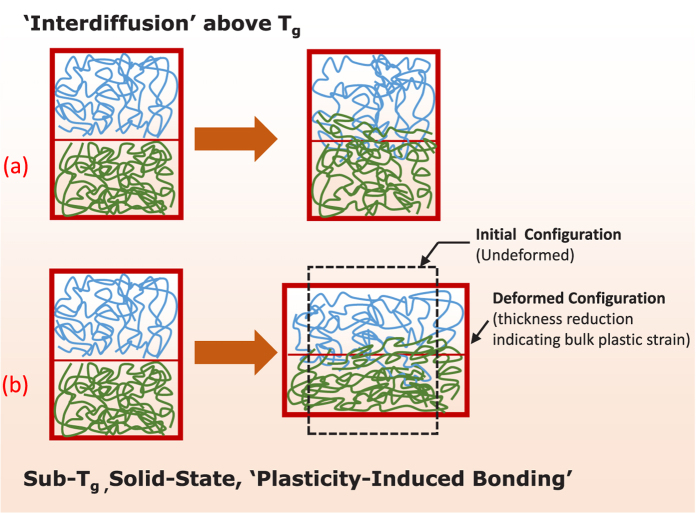
Schematic illustration contrasting mechanisms of interface molecular interpenetration (**a**) Polymer self-adhesion generated by molecular diffusion at temperatures near or above T_*g*_. (**b**) Newly-proposed sub-T_*g*_, solid-state, plasticity-induced bonding in which bulk plastic deformation triggers the requisite molecular mobility for chain interpenetration across the interfaces.
