# Supplementary material for: A New Phenomenon: Sub-Tg, Solid-State, Plasticity-Induced Bonding in Polymers
Source: Sci Rep. 2017 Apr 20;7:46405. doi: 10.1038/srep46405 (PMC5397850; doi:10.1038/srep46405)
Supplement: Supplementary Information [file srep46405-s8.pdf]

Supplementary Material for A New Phenomenon: Sub-T_g, Solid-State, Plasticity-Induced Bonding in Polymers

Nikhil Padhye, David M. Parks, Bernhardt L. Trout, Alexander H. Slocum

1 SUPPLEMENTARY FILM-MAKING

Table 1: **Formulations employed in making polymer films from HPMC E3 and E15 with different levels of plasticizer (the amounts have been rounded off to nearest grams).**

Polymer film	Composition				
	E3 (g)	E15 (g)	Water (g)	EtOH (g)	PEG (g)
E3/E15 in 1:1-0% PEG	15	15	96	96	0
E3/E15 in 1:1-28.5% PEG	15	15	96	96	12
**E3/E15 in 1:1-42.3% PEG**	**15**	**15**	**96**	**96**	**22**
E3/E15 in 1:1-59.5% PEG	15	15	96	96	44
E3-alone-42.3% PEG	30	0	96	96	22
E15-alone-42.3% PEG	0	30	96	96	22

Table 1 shows the sample weights of the contents used in preparation of the solutions. As seen in Table 1, films are referred to based on the amounts of E3, E15 and Wt.% of PEG-400. For example, E3/E15 in 1:1-42.3% PEG implies that E3 and E15 are present in one-to-one ratio and the Wt.% of PEG in the film is 42.3%, since 22 g PEG in 15 g E3 plus 15 g E15 is 42.3%.

Karl Fischer titration was carried out to determine the residual moisture content in the films after drying. Dimethyl sulfoxide (DMSO) was purchased from Sigma Aldrich (ACS reagent grade) and used as a reagent for dissolving films.The solution of the dissolved film in DMSO was fed into the Karl Fischer Titrator, and the residual moisture was estimated. Table 2 shows the average amounts (repeated three times) of the estimated residual moisture contents in the films.

Table 2: **Residual moisture in films after drying, measured through Karl Fischer titration. %Wt. indicates residual moisture in the films after drying.**

**Polymer film**	**Residual H_2O** (%Wt.)
E3/E15 in 1:1-0% PEG	3.70
E3/E15 in 1:1-28.5% PEG	7.21
E3/E15 in 1:1-42.3% PEG	4.29
E3/E15 in 1:1-59.5% PEG	2.45
E3-alone-42.3% PEG	2.92
E15-alone-42.3% PEG	4.54

2 SUPPLEMENTARY CHARACTERIZATION

2.1 Role of Plasticizer and Mechanical Properties

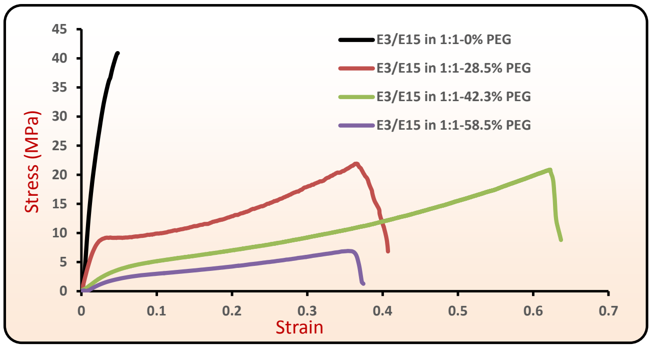

(a)

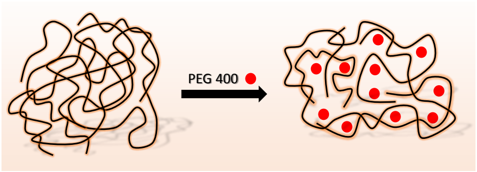

(b)

Figure 1: (a) Effect of PEG-400 on tensile true stress-strain behavior of polymeric films. The tensile tests were carried out at ambient temperature and a nominal strain rate of 0.0025 s^{-1}. (b) Schematic role of plasticizer on molecular configurations.

Figure 1(a) shows true stress-strain behavior of films made from E3/E15 in 1:1 with varying the PEG concentrations 0%, 28.5%, 42.3% and 59.5%, respectively. The

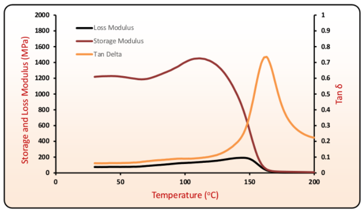

(a) E3/E15 in 1:1-0% PEG

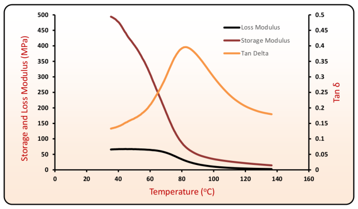

(b) E3/E15 in 1:1-28.5% PEG

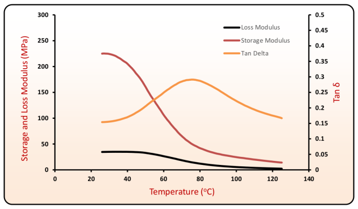

(c) E3/E15 in 1:1-42.3% PEG

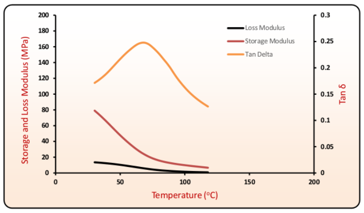

(d) E3/E15 in 1:1-58.5% PEG

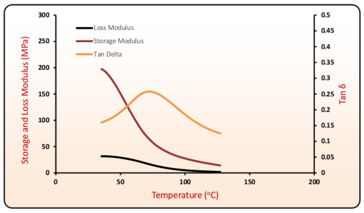

(e) E3-alone-42.3% PEG

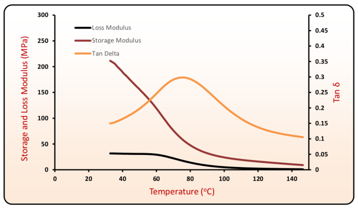

(f) E15-alone-42.3% PEG

Figure 2: Dynamic Mechanical Analysis Curves.

plasticization effect of increasing PEG (Wt.%) is evidenced by the lowering of the initial modulus and the yield strength and, increase in the failure strain. The maximum failure strain occurs for 42.3% PEG film. Clearly, all films containing PEG demonstrate large ductility that is absent in 0% PEG film.

2.2 Dynamic Mechanical Analysis

Dynamic Mechanical Analysis was performed on all the films listed in Table 1. A temperature sweep was performed at 1 Hz frequency. Figures 2(a) to 2(f), show the plots of loss modulus, storage modulus and tan δ for six different films. The glass transition temperature is determined by the peak in the tan δ. For E3-alone-42.3% PEG T_g was estimated to be 72° C, and for E15-alone-42.3% PEG and E3/E15 in 1:1-42.3% PEG T_g was estimated as 78° C. Inclusion of PEG evidently lowers the glass transition temperature and broadens the temperature range over which the glass transition takes place.

2.3 Molecular Weight

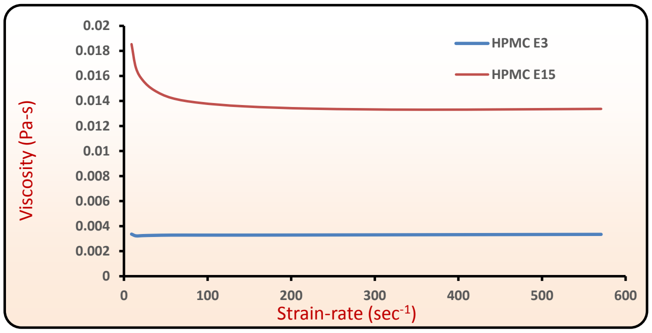

Figure 3: Viscosity curves of 2% aqueous solution of METHOCEL-E3 and METHOCEL-E15.

Viscosity measurements for 2% aqueous solution of E3 and E15 were carried out based on the procedure prescribed by Dow [2]. The viscosity curves for E3 and E15 are shown in the Figure 3. If we choose a representative viscosity of 3.8 mPa-s for E3 and 16 mPa-s for E15, then based on the viscosity and molecular weight relationship from [1], we estimate the number average molecular weight (M_n) for E3 and E15 approximately as 8,200 and 20,000, respectively.

A detailed molecular characterization of METHOCEL cellulose ethers presented in [14], also led to estimation of weight average (M_w) and number average (M_n) molecular weights as: (i) E3: $M_n = 8,100$ and $M_w = 20,300$ with M_w/M_n = 2.5, and (ii) E15: $M_n = 24,800$ and $M_w = 60,300$ with M_w/M_n = 2.4. Such estimations are consistent with those we obtained. In the same study [14], the degree of polymerization (DP) was reported as: (i) E3, DP= 77, and (ii) E15, DP=296, and the weight average radius of gyration (R_{gw}) as: (i) E3, R_{gw} = 7.4 nm, and (ii) E15, R_{gw} = 15.1 nm.

2.4 X-Ray Diffraction

HPMC is a cellulose derivative and well known to exist in amorphous form. As an illustration, XRD pattern of E3/E15 in 1:1-42.3% PEG is shown in Figure 4. As expected, a diffused pattern without any peaks is obtained, thus, indicating absence of any crystallinity.

2.5 Atomic Force Microscopy

Figure 5 shows sample AFM scans of a 5 μm x 5 μm area on the top surface of three films with 42.3% PEG, along with the average roughness given by R_a.The top surfaces of films exhibit nano-scale roughness, however, this scale of roughness does not play any important role when we have reported bulk plastic strains essential for bonding.

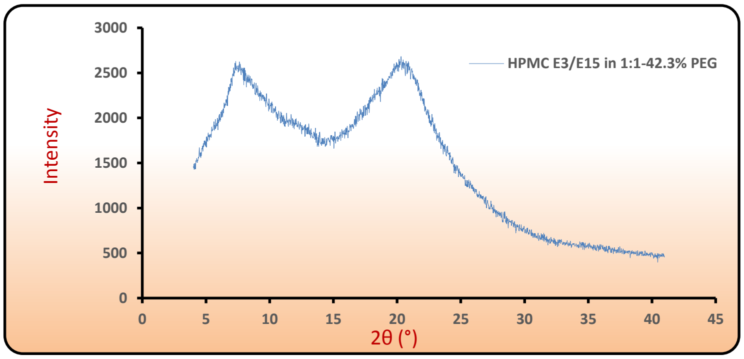

Figure 4: XRD of E3/E15 in 1:1-42.3% PEG.

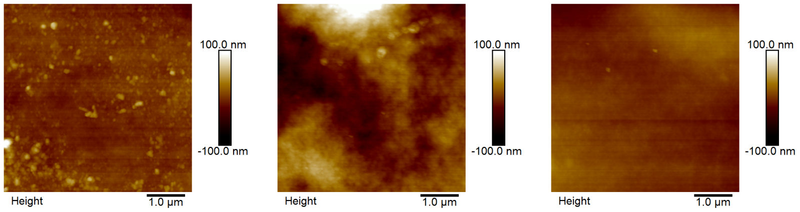

(a) Top surface of E3/E15 in 1:1-42.3% PEG film, $R_a = 6.91$ nm

(b) Top surface of E3-alone-42.3% PEG film, $R_a = 22.7$ nm

(c) Top surface of E15-alone-42.3% PEG film, $R_a = 8.63$ nm

Figure 5: Measurement of Nanoroughness using Atomic Force Microscopy.

3 SUPPLEMENTARY DISCUSSION

3.1 Polymer Dynamics and Self-Diffusion

Polymer melts are an equilibrium system and their mobility is commonly described by the models of Rouse, reptation, etc. The fundamental dynamic property that characterizes the average motion of a polymer chain is the coefficient of self-diffusion (D). Typical values of self-diffusion coefficients from the literature are listed in Table 3.

Table 3: **A Short Summary of Self-Diffusion Coefficient (D) from literature. T_m and T_g stand for melting and glass transition temperature, respectively.**

**Ref.**	**Polymer**	**Mol. Wt.** (g/mol)	**Temp.** (K)	**D** (m^2/s)	**Comments**
[7]	Linear H-PB	5×10^4 – 20×10^4	398.15	10^{-14} – 5×10^{-16}	$T_m \sim 381.15$ K
[10, 8]	Polyisoprene	560 – 9.82×10^4	373.15	2.2×10^{-10} – 1.0×10^{-14}	$T_g \sim 192$ K [3]
[10]	Polybutadiene	690 – 4.99×10^4	373.15	7.0×10^{-11} – 2.0×10^{-14}	$T_g \leq 183.15$ K
[19]	Polyethylene	200 – 12×10^4	448.15	6.6×10^{-10} – 1.3×10^{-14}	$T_m \sim 353.15$ – 400.15 K
[16]	PDMS	500 – 5×10^5	293.65	7.0×10^{-10} – 5×10^{-15}	$T_g \sim 150.15$ K
[9]	PS	600 – 19×10^3	487.8	2.5×10^{-10} – 1.5×10^{-13}	$T_g \sim 333.15$ – 373.15 K

3.2 Stress-Induced Molecular Mobility and Plastic-Deformation

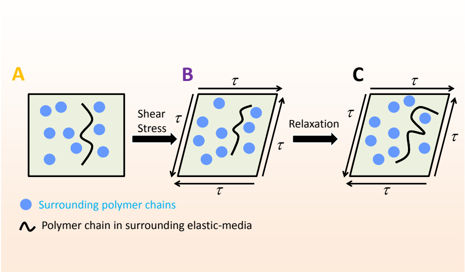

(a)

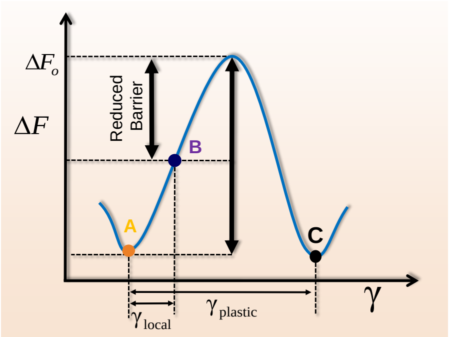

(b)

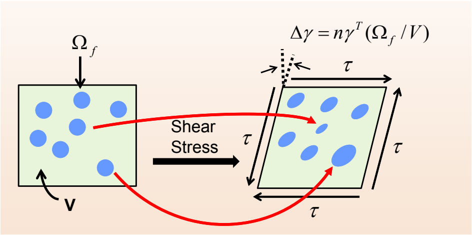

(c)

Figure 6: Mechanism of plastic deformation and shear transformation in glassy polymers [5]. (a) A unit shear transformation in a kinetically trapped state under shear stress comprises of an initial elastic shear-strain which is followed by plastic-relaxation of polymer chain segments. (b) Free energy landscape, associated with a polymer chain, during a unit shear transformation (c) Accumulation of several shear transformations leads to macroscopic plastic deformation.

As stated in the main letter, plastic deformation in polymers at a continuum scale is understood in terms of shear transformations i.e. events of spatial rearrangements of molecular clusters causing stress-relaxation. Consider the scenario shown in the Figure 6: well below T_g, the polymer chains are kinetically trapped in their local configurations and timescales for mobility (specifically translation motions) of these chains are

extremely large. However, application of shear-stress on the material element causes its deformation and the polymer chain under consideration changes its orientation: first elastically, and then due to some local perturbation it relaxes plastically while overcoming the potential barrier set up due to neighboring molecules. Thus, qualitatively speaking, the application of *stress enhances the mobility* of the polymer chain as it relaxes, and changes its configuration on experimental time scales. Effects of such cumulative events during active plastic deformation characterize the enhanced dynamics in deforming glasses below T_g.

3.3 Temperature Rise Due to Plastic Deformation

As an illustration, we measured the specific heat of E3/E15 in 1:1-42.3%PEG film through differential scanning calorimetry, as shown in Figure 7. From the rate of heat flow into the sample and specified rate of temperature rise during thermal scan the C_p is obtained as 1860 J/Kg-K, and the density was measured to be $\rho = 1180$ Kg/m^3. Based on the stress-strain curves, if we estimate the flow stress for plastic deformation to be $\sigma_f = 8$ MPa, then for a plastic strain of $\varepsilon_p = 0.5$, a simple estimate of adiabatic temperature rise is:

$$\triangle T = \frac{\sigma_f \varepsilon_p}{\rho C_p} = 3.6^{\circ}C.$$

As seen here, the temperature rise, even according to fully adiabatic analysis, is quite small. External work due to the application of stresses leads to mechanically-assisted (and not temperature assisted) enhanced mobility of polymer chains (or segments).

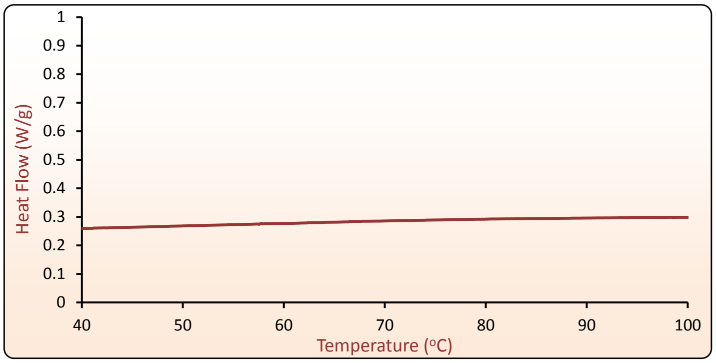

Figure 7: DSC scan of E3/E15 in 1:1-42.3% PEG film.

4 Bonding Experiments

4.1 Roll-Bonding Machine

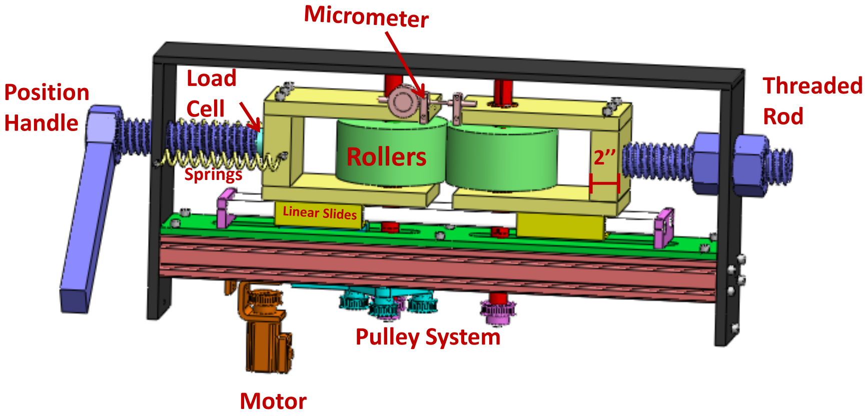

Figure 8: A roll-bonding machine to carry out sub-T$_g$, solid-state, plasticity-induced bonding. For more details see [17].

Figure 8 shows a CAD model of the roll-bonding machine designed for this work. The machine is capable of achieving different levels of plastic strain by adjustment of the gap between the rollers and monitoring the compression load during rolling. The angular speed of the rollers is controlled using a stepper motor. The radius of the rollers (R) is 100 mm, much larger than the total initial thickness of a film-stack (t_1), which is typically less than 1 mm. The incoming stack of film behaves like a thin strip and through-thickness plastic deformation is triggered under such conditions. From kinematics of rigid-plastic rolling of thin-strip [12] the time spent during active plastic deformation can be estimated as follows:

$$\tau = \frac{\sqrt{R(t_1 - t_2)}}{V_2}. \tag{1}$$

In the above equation, t_1 is the initial thickness of film-stack, t_2 is the thickness of film-stack at the exit, V_2 is the linear speed at the exit. For V_2 = 5.23 mm/s, t_1=0.6 mm and t_2=0.45 mm (indicating 25% nominal plastic strain), the time spent by a material element under the roller would be approximately 0.74 s. This is how we achieve sub-T$_g$, solid-state, plasticity-induced roll-bonding in a period of time on the order of a second. The Supplementary video S1 demonstrates how a stack of films with a certain initial thickness is subjected to active plastic straining leading to sub-T$_g$, solid-state, plasticity-induced bonding. The final thickness of the roll-bonded stack is less than the initial. Complete details on the roll-bonding machine and process are available in [17].

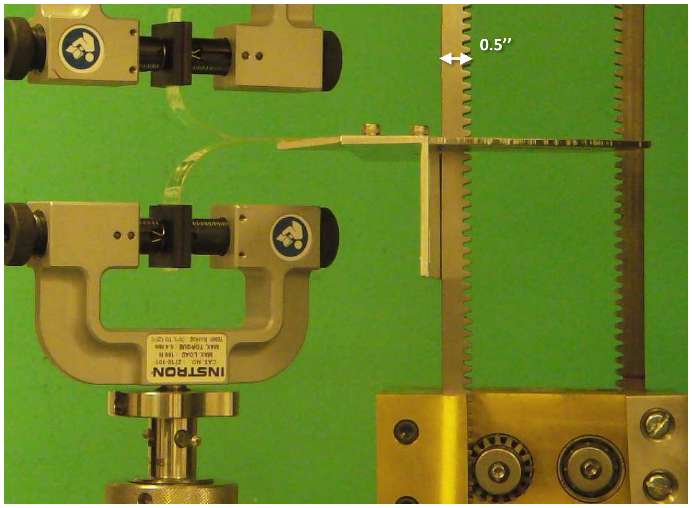

Figure 9: Peel-Test in mechanical tester to determine mode-I fracture toughness (G_c).

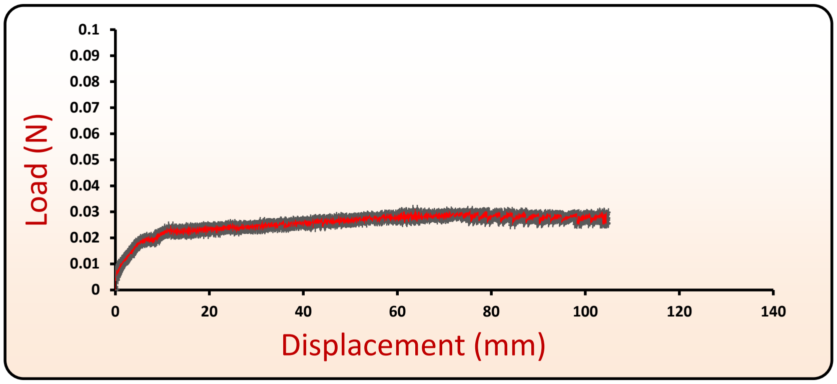

Figure 10: Force versus displacement curve during Peel-Test. In the steady-state peeling the peel-force becomes steady with respect to cross-head displacement.

Table 4: **For a given exit speed (V_2 = 5.23 mm/sec) and an initial thickness t_1 = 0.6 mm, estimates of time spent under the roller bite during plastic straining.**

**Plastic Strain**	**t_2** (mm)	**Time** (seconds)
0.05	0.57	0.33
0.1	0.54	0.47
0.15	0.51	0.57
0.20	0.48	0.66
0.25	0.45	0.74

4.2 Peel Test

Figure 9 shows a snapshot of the peel test. A peel test fixture was designed to perform accurate mode-I fracture testing. Such a test is also commonly known as T-peel test in the literature. The designed fixture [17, 18], provides support to a long tail of the peel-specimen and eliminates any spurious effects due to gravity. The Supplementary video S4 gives an illustration on how the peel tests were conducted (also see [18] for more details).

When a stack of six layers is roll-bonded, a total of five bonded interfaces are formed. Peeling is done at the central interface. Figure 10 shows force versus displacement curve during a peel test. For all peel tests a cross-head speed of 15 mm/min was chosen. The steady-state peeling force P is used to estimate the rate of external work per unit advance of crack as 2P/b, where 'b' is the width of the specimen (typically 15 – 20 mm). In order to correctly determine the fracture toughness (G_c) of the plastically-welded interface, any amount of plastic work due to bending of peel arms must be subtracted from the total steady state work [15].

Since glassy polymers may exhibit both kinematic and isotropic hardening, we measured their yield strength in tension after the roll-bonding (at different levels of bonding strain). As an illustration, Figure 11 shows the true stress-strain curves in tension for E15-alone-42.3% PEG films roll-bonded at different levels of nominal plastic strain. It is seen that yield points of films in tension after roll-bonding at different levels of plastic strain are not much different than the yield point of the starting film. This indicates that effect of plastic strain, during roll-bonding, on the yield strength of the films is negligible, and therefore there is no special need to use a modified yield strength for analyses in mechanics of peel test. A detailed illustration is provided in [17]. The error bars in G_c (as shown in the main letter) are based on the variation when peeling force becomes steady.

4.3 Lap-Shear Testing

Preparation of lap specimens and shear-strength measurements were carried out in Instron testing machine. A lap joint was assembled between two film layers, each layer being nearly 100 μm thick. The overlapping region was nearly $A = 5 \times 5 = 25$ mm^2 in area. A cross-head speed of 0.5 mm/min was chosen to apply desired compression load

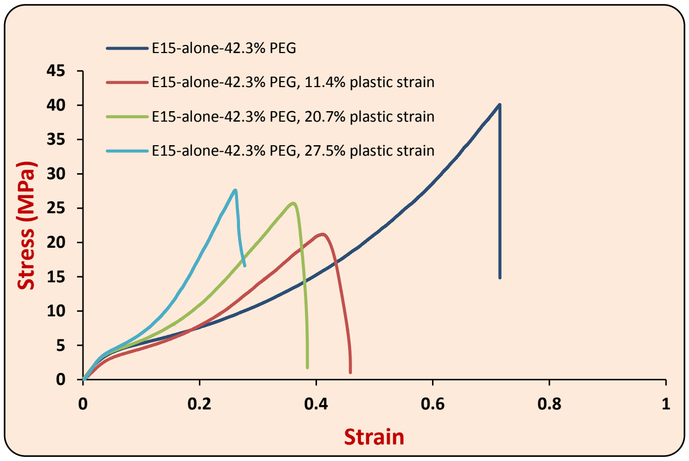

Figure 11: True stress-strain curves under tension for E15-alone-42.3% PEG, after roll-bonded at different levels of nominal plastic strain.

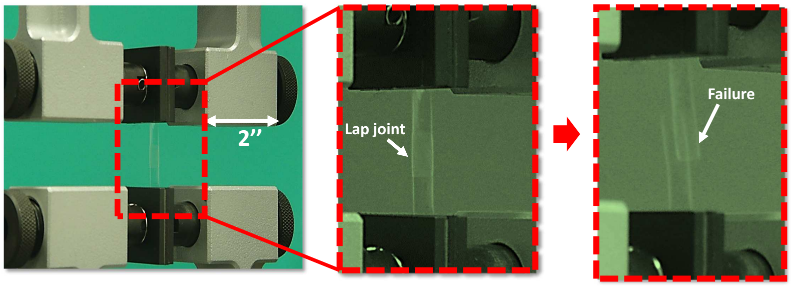

Figure 12: Lap shear-strength test specimen in tensile tester.

on the overlapping area. The sample was plastically bonded by pressing between two parallel (accuracy: 1 μm) flats. Lap joint was tested for shear-strength in tension mode (at a cross-head speed of 15 mm/min). A snapshot of the test is shown in Figure 12. The peak force before failure divided by the bonded area was taken as the lap shear-strength. Figure 13 shows the force versus displacement during a lap shear-strength measurement.

4.4 Mechanics of Axisymmetric Upsetting

Unconstrained compression of a film stack with initial thickness much smaller than the radius of the stack qualifies as a classic case of an upsetting problem. As shown in the Supplementary video S2-a, a film stack with an initial thickness of 0.84 mm requires peak loads up to 40 kN (which equates to a peak nominal stress of 78.9 MPa, much larger than yield strength of the polymer) in order to achieve a final thickness of 0.70

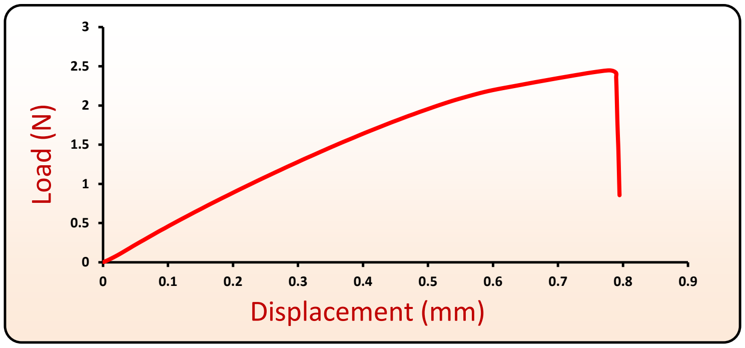

Figure 13: Force versus displacement curve during lap shear-strength testing. For this specimen a nominal plastic strain of 9.0% was imposed on an overlapping area of 5 mm by 5 mm and the shear-strength (σ_s=F$_{max}$/A) was estimated to be 0.07 MPa.

mm due to compression. This can be attributed to radially-inward directed frictional forces acting on the top and bottom surfaces during compression.

Figure 14 schematically shows the upsetting of a film stack. Figure 15 shows the stress components along with the frictional forces acting on a cylindrical element.

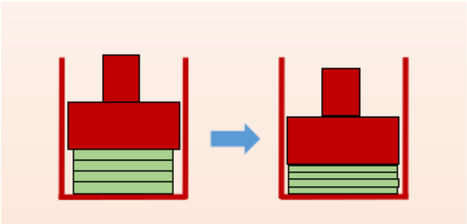

Figure 14: Axisymmetric Upsetting of laminates.

Here, we employ the upper bound analysis to predict the loads required to achieve plastic deformation in upsetting scenario and compare it with the experimentally noted loads. The analysis presented here is borrowed from [4, 13], where a detailed derivation can also be found.

If $\dot{E}_T$ is the total energy rate expended during material deformation, then $\dot{E}_T = L \times V$ [6], where L represents the forming load and V represents the velocity of the die. The total energy expended in material deformation itself can be expressed as sum of energy rates for deformation ($\dot{E}_D$) and frictional work ($\dot{E}_F$):

$$\dot{E}_T = \dot{E}_D + \dot{E}_F \tag{2}$$

The deformation energy rate is given as a volume integral of dissipation:

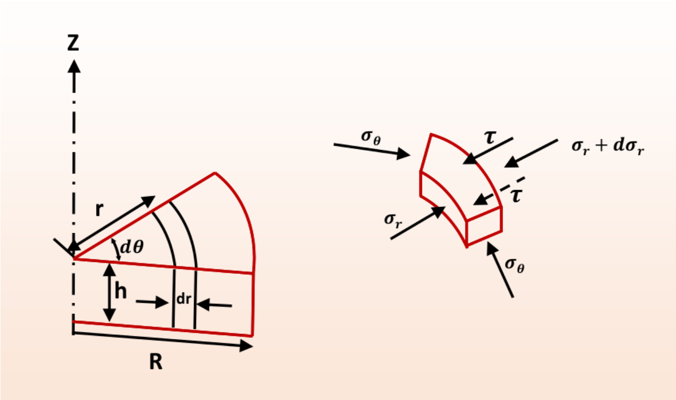

Figure 15: Stresses and frictional forces acting on an element during axisymmetric upsetting.

$$\dot{E}_D = \int_V \bar{\sigma}\dot{\bar{\varepsilon}} dV \tag{3}$$

where $\bar{\sigma}$ is the flow stress for plastic deformation and $\dot{\bar{\varepsilon}}$ is the effective strain rate. $\dot{\bar{\varepsilon}}$, in the present scenario of homogeneous axisymmetric deformation, is equal to V_D/h (with V_D being the speed of the die during compression and h the height of the stack at a particular instant). The rate of work due to frictional dissipation ($\dot{E}_F$) includes the friction energies on both the top and bottom surface of the deforming part and is given as an integral of dissipation over the surfaces:

$$\dot{E}_F = 2\int_{SF} \tau v ds \tag{4}$$

where τ and $v = (V_D \cdot 2\pi r/2h)$ denote the shear stress and radial velocity on the top and bottom surfaces. The shear stresses τ is assumed to be equal to $m\bar{\sigma}/\sqrt{3}$ (with m being the friction factor). Computing the quantities in equations (3) and (4) and substituting them into (2), we arrive at the total energy rate:

$$\dot{E}_T = \pi R^2 \bar{\sigma} V_D + \frac{2}{3}\pi m \frac{\bar{\sigma}}{\sqrt{3}} \frac{V_D}{h} R^3.$$

Thus, the load is estimated as:

$$L = \frac{\dot{E}_T}{V_D} = \pi R^2 \bar{\sigma} \left(1 + \frac{2}{3\sqrt{3}} m \frac{R}{h}\right). \tag{5}$$

During upsetting $t_o = 0.84$ mm, $t_f = 0.7$mm and therefore $e = ln(t_o/t_f) \approx 0.18$, and loading occurred at a crosshead speed of 6 mm/min (leading to an estimate of nominal strain rate of 0.13 sec^{-1}). Figure 16 shows the strain-rate sensitivity measurements in tension; from which a flow stress of $\bar{\sigma} = 10$ MPa is estimated. Based on the assumption of volume conservation during upsetting, the final radius (R) corresponding to $t_f = 0.7$

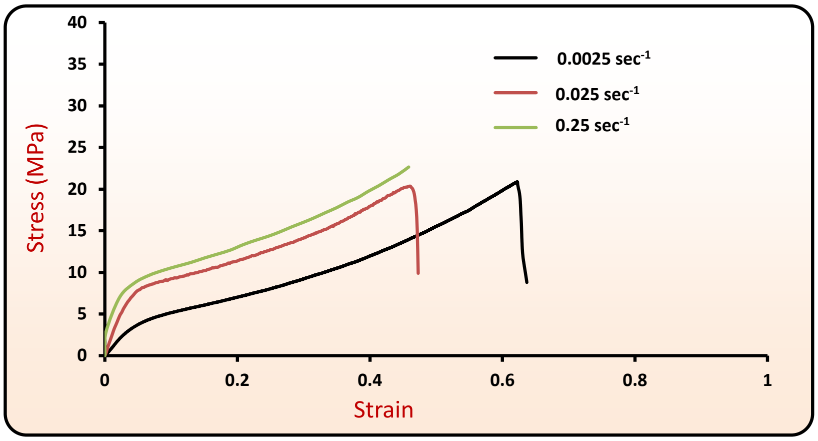

Figure 16: Strain-rate sensitivity of E3/E15 in 1:1-42.3% PEG. Nominal strain-rates are listed.

mm is estimated as 13.7 mm. By choosing an extreme value of $m = 1$ and substituting other quantities in equation (5), we estimate the upper bound load $L = 50.29$ kN. Thus, it is clear that the friction plays a significant role, despite the use of Teflon, in enhancing the loads required for bulk plastic deformation in 'upsetting' (where $R/h >> 1$).

4.5 Mechanics of uniaxial strain die

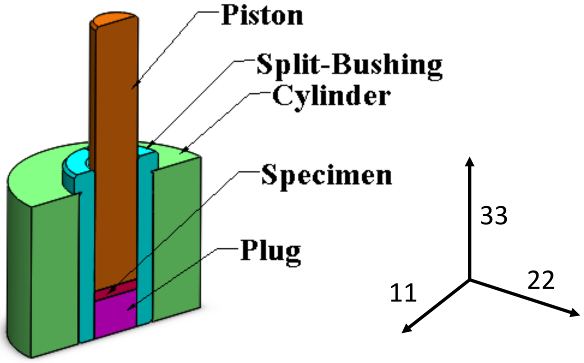

Figure 17: A set-up to achieve uniaxial strain in compression.

As discussed in the main letter and Supplementary video S2, the purpose of designing a 'uniaxial strain die' was to explicitly show the role of active plastic deformation in achieving sub-T_g, solid-state, plasticity-induced bonding. Figure 17 shows the CAD model of a 'uniaxial strain die'. Such a setup is capable of generating large levels of

hydrostatic pressure, while strongly limiting the plastic flow to negligible levels, when a circular stack of film with a radius equal to the internal radius of the cavity is compressed inside the die.

We present a couple of analyses to demonstrate the principle of the 'uniaxial strain die'. Illustrations related to deformation theory of plasticity, as presented here, are borrowed in parts from [11, 13]. In what follows next, a boldface letter is to used to indicate a tensor variable.

4.5.1 Elasticity Analysis

First, we consider axisymmetric elastic compression of a film stack placed in the die. Here, all strains are assumed to be elastic and frictional forces are assumed to be absent. The solution to this problem is derived from the standard procedure of stresses in a thick-walled cylinder with a zero internal radius. A cylindrical coordinate system (r, Θ, z) is used. The principal stress components are denoted by σ_r, σ_Θ and σ_z, and the associated strains given as ε_r, ε_Θ and ε_z. All other shear components are zero. Due to axisymmetry and wall constraints inside the die we have $\sigma_r = \sigma_\Theta$ $(= \sigma$, say) and $\varepsilon_r = \varepsilon_\Theta = 0$. Using the boundary constraints with the stress-strain relation of linear elasticity:

$$\boldsymbol{\varepsilon} = \frac{1+\nu}{E}\boldsymbol{\sigma} - \frac{\nu}{E}tr(\boldsymbol{\sigma})\mathbf{I}, \tag{6}$$

we find,

$$\sigma_z = \frac{E(1-\nu)\varepsilon_z}{(1-2\nu)(1+\nu)} \tag{7}$$

$$\sigma = \frac{E\nu\varepsilon_z}{(1-2\nu)(1+\nu)} \tag{8}$$

where, E and ν are Young's modulus and Poisson's ratio, respectively. The stress tensor in terms of principal directions $\bar{e}_r$, $\bar{e}_\Theta$ and $\bar{e}_z$ is given as $\boldsymbol{\sigma} = \sigma\, \bar{e}_r \otimes \bar{e}_r + \sigma\, \bar{e}_\Theta \otimes \bar{e}_\Theta + \sigma_z\, \bar{e}_z \otimes \bar{e}_z$. $\boldsymbol{\sigma}$ can be decomposed into deviatoric part ($\boldsymbol{\sigma}'$) and hydrostatic part ($\sigma_m\mathbf{I}$, with $\sigma_m = (\sigma_r + \sigma_\Theta + \sigma_z)/3$ denoting the mean normal stress), and written as $\boldsymbol{\sigma} = \boldsymbol{\sigma}' + \sigma_m\mathbf{I}$. The von Mises stress ($\sigma_v$) is given as:

$$\sigma_v = \sqrt{\frac{3}{2}\boldsymbol{\sigma}' : \boldsymbol{\sigma}'} \tag{9}$$

Thus, in the presence of die the von Mises stress ($\sigma_{v,die}$) and hydrostatic pressure ($p_{die} = -\sigma_m$) are given as:

$$p_{die} = \frac{E|\varepsilon_z|}{3(1-2\nu)} \tag{10}$$

$$\sigma_{v,die} = \frac{E|\varepsilon_z|}{1+\nu} \tag{11}$$

In contrast if we imagined axisymmetric, unconstrained elastic compression, without any frictional effects then we would have $\sigma_z = E\varepsilon_z$, and $\sigma_r = \sigma_\Theta = 0$. In such case the von Mises stress ($\sigma_{v,no-die}$) and hydrostatic pressure (p_{no-die}) would be given as:

$$p_{no-die} = \frac{E|\varepsilon_z|}{3} \tag{12}$$

$$\sigma_{v,no-die} = |\sigma_z| \tag{13}$$

We emphasize that elasticity analysis is valid only up to the onset of plastic deformation. However, if we are within the elastic limit then equations (10) and (12) show that large hydrostatic-pressure can build up during compression inside the die. Particularly in the limit as $\nu \to 0.5$, $p_{die} \to \infty$.

In the video S2-b part II, a maximum compressive load of 40 kN is applied on a film-stack with radius $0.5'' = 12.5$ mm; corresponding to $\sigma_z = -78.98$ MPa. According to equation (7), if $\sigma_z = -78.98$ MPa, $\nu = 0.45$, and E=300 MPa (approximated from the nominal strain rate of 0.13 sec^{-1}, see Figure 16), then $\varepsilon_z \approx -0.12$. Substituting $|\varepsilon_z| = 0.12$, $E = 300$ MPa and $\nu = 0.45$ in equation (11), $\sigma_{v,die} = 25.7$ MPa. Clearly, $\sigma_{v,die}$ thus obtained is larger than the yield strength of the film. This indicates the possibility of plastic deformation even in the presence of the die. Next, we present an analysis based on the incremental (or "flow theory") of plasticity, which accounts for plastic deformation.

4.5.2 Incremental ("Flow Theory") of Plasticity

Here, we take into account the plastic deformation and demonstrate how little amount of plastic straining occurs when a film stack is compressed in the presence of the 'uniaxial strain die' and is loaded to values of $|\sigma_z| >> \sigma_{yield}$.

The total strain increment tensor ($d\boldsymbol{\varepsilon}$) is taken as the sum of the elastic strain increment tensor ($d\boldsymbol{\varepsilon}^e$) and the plastic strain increment tensor ($d\boldsymbol{\varepsilon}^p$):

$$d\boldsymbol{\varepsilon} = d\boldsymbol{\varepsilon}^e + d\boldsymbol{\varepsilon}^p \tag{14}$$

The increment in elastic strain tensor can be derived using equation (6) and written as:

$$d\boldsymbol{\varepsilon}^e = \frac{1+\nu}{E} d\boldsymbol{\sigma}' + \frac{1-2\nu}{E} d(tr(\boldsymbol{\sigma}))\mathbf{I} \tag{15}$$

Under multi-axial loading the behavior of ductile materials can be described by the Levy-Mises equations, which relate the principal components of strain increments in plastic deformation to the principal applied stresses (deviatoric components). In the present scenario this can be expressed as:

$$\frac{\dot{\varepsilon_r}^p}{\sigma'_r} = \frac{\dot{\varepsilon_\Theta}^p}{\sigma'_\Theta} = \frac{\dot{\varepsilon_z}^p}{\sigma'_z} \tag{16}$$

On the grounds of axisymmetric compression, similar to what was discussed in the previous section, we have $\sigma_r = \sigma_\Theta$ ($= \sigma$, say), and therefore $\dot{\varepsilon}_r = \dot{\varepsilon}_\Theta$ ($\dot{\varepsilon}$, say).

We further define following quantities to aid this illustration:

$$|\boldsymbol{\sigma}'| = \sqrt{\boldsymbol{\sigma}' : \boldsymbol{\sigma}'} \tag{17}$$

$$\bar{\sigma} = \sqrt{\frac{3}{2}\boldsymbol{\sigma'} : \boldsymbol{\sigma'}} = \sqrt{\frac{3}{2}}|\boldsymbol{\sigma'}| \tag{18}$$

$$|d\boldsymbol{\varepsilon}^p| = \sqrt{d\boldsymbol{\varepsilon}^p : d\boldsymbol{\varepsilon}^p} \tag{19}$$

$$d\bar{\varepsilon}^p = \sqrt{\frac{2}{3}d\boldsymbol{\varepsilon}^p : d\boldsymbol{\varepsilon}^p} = \sqrt{\frac{2}{3}}|d\boldsymbol{\varepsilon}^p| \tag{20}$$

The flow rule can be written as:

$$\frac{d\boldsymbol{\varepsilon}^p}{|d\boldsymbol{\varepsilon}^p|} = \frac{\boldsymbol{\sigma'}}{|\boldsymbol{\sigma'}|} \tag{21}$$

The total increment in plastic strain can be written as:

$$d\boldsymbol{\varepsilon}^p = -\frac{1}{2}d\varepsilon_z^p \bar{e}_r \otimes \bar{e}_r - \frac{1}{2}d\varepsilon_z^p \bar{e}_\Theta \otimes \bar{e}_\Theta + d\varepsilon_z^p \bar{e}_z \otimes \bar{e}_z \tag{22}$$

It is worth noting that at any instance neither the directions nor the relative magnitudes of plastic strain components change, and therefore we can write equation (22) as:

$$\boldsymbol{\varepsilon}^p = -\frac{1}{2}\varepsilon_z^p \bar{e}_r \otimes \bar{e}_r - \frac{1}{2}\varepsilon_z^p \bar{e}_\Theta \otimes \bar{e}_\Theta + \varepsilon_z^p \bar{e}_z \otimes \bar{e}_z \tag{23}$$

By choosing $\boldsymbol{D} = -\frac{1}{2}\bar{e}_r \otimes \bar{e}_r - \frac{1}{2}\bar{e}_\Theta \otimes \bar{e}_\Theta + \bar{e}_z \otimes \bar{e}_z$, we can re-write equation (23) as:

$$\boldsymbol{\varepsilon}^p = \varepsilon_z^p \boldsymbol{D} \tag{24}$$

In equation (24), ε_z^p is negative during compression. Using the flow rule from equation (21), and the fact that increments in plastic strains are proportional to the principal directions (which are constant throughout the deformation history); we can rewrite flow rule in terms of total plastic strain at any instant as:

$$\frac{\boldsymbol{\varepsilon}^p}{|\boldsymbol{\varepsilon}^p|} = \frac{\boldsymbol{\sigma'}}{|\boldsymbol{\sigma'}|} \tag{25}$$

If we assume plastic deformation to continue at a constant yield Y, then:

$$Y = \sqrt{\frac{3}{2}}|\boldsymbol{\sigma'}| \tag{26}$$

It is worth mentioning that the yield strength of polymers is usually a function of hydrostatic pressure and plastic strain (and the flow stress increases with increasing hydrostatic pressure and plastic strain, thus an assumption of constant yield stress is an under estimation).

By combining equations (24), (25) and (26) we can write:

$$\boldsymbol{\sigma'} = \frac{\varepsilon_z^p}{|\varepsilon_z^p|}\frac{2}{3}Y\boldsymbol{D} \tag{27}$$

Since ε_z^p is negative during compression, equation (27) can be re-written as:

$$\boldsymbol{\sigma}' = -\frac{2}{3}Y\boldsymbol{D} \tag{28}$$

The elastic strain increments, as given in equation (15), occur both due to deviatoric stress and hydrostatic stress. At the onset of plastic flow (and continued plastic yielding at constant flow stress) the the deviatoric stress becomes constant (given by equation (27)), after which there is no further contribution to elastic strains due to deviatoric stress components. However, the normal stress and the hydrostatic part of the elastic strains continue to increase. Thus, total elastic-strain can be written as:

$$\boldsymbol{\varepsilon}^e = -(\frac{1+\nu}{E})(\frac{2Y}{3})\boldsymbol{D} + \frac{\sigma_m}{3}(\frac{1-2\nu}{E})\mathbf{I} \tag{29}$$

From equation (14), the total strain can be written as:

$$\boldsymbol{\varepsilon} = \varepsilon_z^p\boldsymbol{D} - (\frac{1+\nu}{E})(\frac{2Y}{3})\boldsymbol{D} + \frac{\sigma_m}{3}(\frac{1-2\nu}{E})\mathbf{I} \tag{30}$$

Now imposing the constraint that total strains in the r and Θ direction are zero i.e. $\varepsilon_r = \varepsilon_\Theta = 0$ then equation (30) implies:

$$\frac{\sigma_m}{3} = \frac{E}{1-2\nu}[\frac{\varepsilon_z^p}{2} - \frac{Y(1+\nu)}{3E}] \tag{31}$$

Thus, the overall stress tensor can be written as:

$$\boldsymbol{\sigma} = -\frac{2Y}{3}\boldsymbol{D} + \frac{E}{2(1-2\nu)}\varepsilon_z^p\mathbf{I} - \frac{Y(1+\nu)}{3(1-2\nu)}\mathbf{I} \tag{32}$$

If we again choose σ_z = -78.89 MPa (and same elastic constants as used in the previous section), we now get the much-reduced estimate $|\varepsilon_z^p| \approx 0.023$. We emphasize the fact that in polymers the plastic straining is accompanied with hardening, and the yield stress increases with mean normal pressure, therefore, $|\varepsilon_z^p| = 0.023$ is an overestimation of axial plastic strain from this loading of the 'uniaxial strain die', even though the applied axial stress was more than an order of magnitude greater than the yield strength.

Lastly, the ratio $\frac{\sigma_r}{\sigma_z} = 0.96$ which suggests that the state of stress inside the die is 'hydrostatic'.

These calculations demonstrate that despite the large hydrostatic pressures there is only small plastic straining, due to which no bonding outcome is noted.

5 Supplementary Results

Here we present additional and supporting experimental data (G_c and lap shear-strength results) on three films made from E3-alone-42.3% PEG, E3/E15 in 1:1-42.3% PEG and E15-alone-42.3% PEG, and report reproducible trends on the bonding results. Since each set of bonding experiment has been carried out on films made from a particular batch of raw materials and solvents obtained commercially, their mixing, and drying

via solvent casting, we refrain from merging all the data owing to any inherent variation that may be present due to these factors. Figures 18(a) and 18(b) show G_c trends for the three films based on second and third trials corresponding to the two additional batches of material films prepared. Similarly, Figures 19(a) and 19(b) show lap shear-strength measurements for bonding experiments based on second and third batches of materials. Excellent reproducibility in the bonding trends and non-monotonic correlation of G_c and lap shear-strength with plastic strain is noted, and conclusively establishes the new phenomenon of sub-T_g, solid-state, plasticity-induced bonding.

We also report a sample result showing the effect of strain-rate in rolling experiments on films made from E3/E15 in 1:1-42.3% PEG. Two roller speeds of 0.5 rev/min and 0.05 rev/min, leading to exit speeds of 5.23 mm/s and 0.523 mm/s respectively, were selected to study the effect of strain-rate on roll-bonding. Since the time spent in rolling inversely depends on the exit speed (equation (1)), an order of magnitude difference in the exit speeds would approximately amount to an order of magnitude difference in rolling-times, and thus leading to an order of magnitude difference in nominal strain-rate (given by $(t_1 - t_2)/\tau$). G_c with respect to plastic strain for two roller speeds is shown in Figure 20. We observe negligible effect of strain-rate on fracture toughnesses.

At slow to moderate strain-rates, when bonding processes are primarily isothermal then negligible influence of strain-rate can be expected, because whenever adiabatic effects (or any other factors) do not alter the micro-mechanism of plastic deformation that leads to enhanced molecular mobility and interpenetration of polymer chains across the interface, then bonding is likely to depend directly on the gross plastic strain. Warren and Rottler [20] showed that accelerated dynamics in a deforming glass, under different deformation settings which reflected varying strain-rates, directly correlated with net plastic strain.

6 Information on Supplementary Videos

Supplementary video S1: Sub-T_g, solid-state, plasticity-induced roll-bonding of polymeric films.

Supplementary video S2-a: Sub-T_g, solid-state, plasticity-induced bonding of film-stack during compression in upsetting experiment.

Supplementary video S2-b part I: Design of a ‘uniaxial-strain die’ (i.e. ‘hydrostatic die’).

Supplementary video S2-b part II: Compression of film-stack in ‘uniaxial-strain die’.

Supplementary video S2-b part III: No-bonding outcome of film-stack after compression in ‘uniaxial-strain die’.

Supplementary video S3: No-bonding outcome between an ‘elastic’ and ‘plastic’ film.

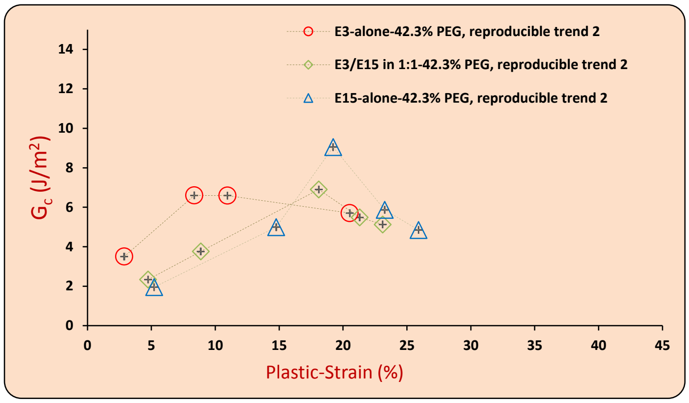

(a)

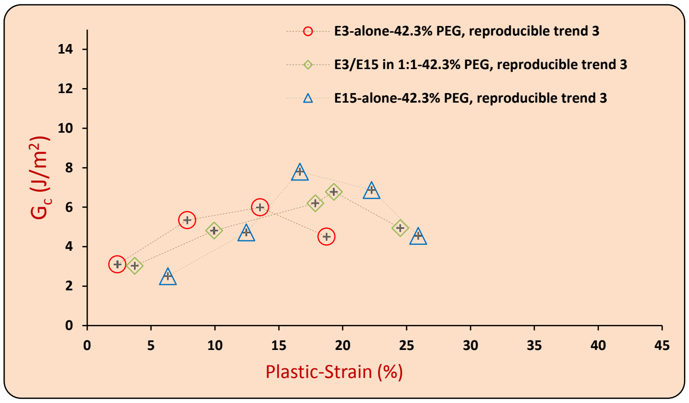

(b)

Figure 18: Reproducible trends for roll-bonding for three films, G_c with respect to plastic strain for second and third batch of materials prepared. G_c is calculated based on the mean steady-state peel force during peeling, and error bars in G_c are based on one standard deviation from fluctuations in the peeling force in steady-state regime. Plastic-strain is calculated based on mean thicknesses before and after bonding (10 measurements), and the error bars in plastic strain are derived from these measurements.

Supplementary video S4: Peel-test on roll-bonded laminates.

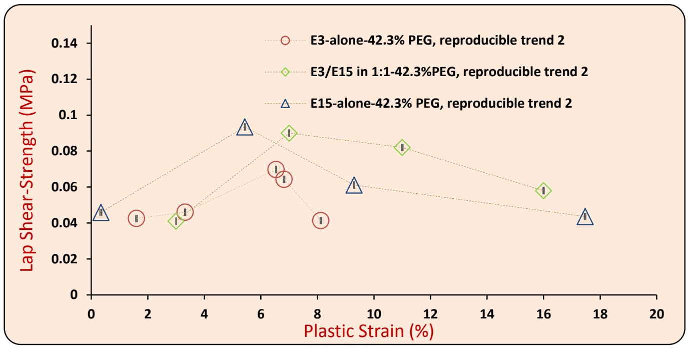

(a)

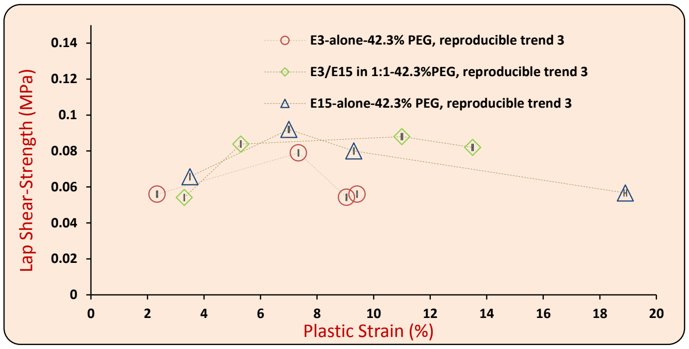

(b)

Figure 19: Reproducible trends for lap shear-strength (σ_s [MPa]) with respect to plastic strain. Plastic-strain is calculated based on mean thicknesses before and after bonding (10 measurements), and the error bars in plastic strain are derived from these measurements.

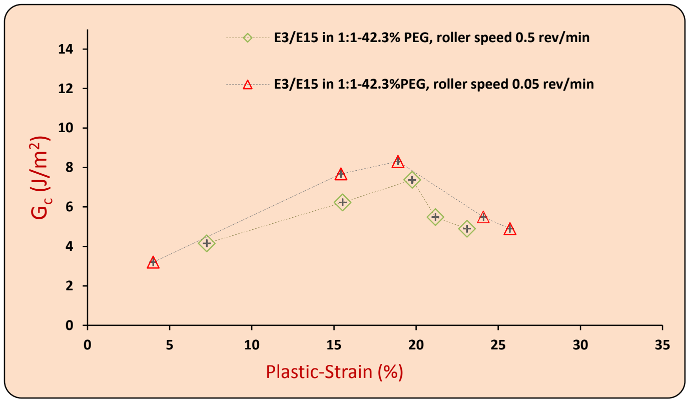

Figure 20: Effect of strain-rate on G$_c$ with respect to plastic strain.

References

[1] Methocel cellulose ethers in aqueous systems for tablet coating. http://www.dow.com/scripts/litorder.asp?filepath=/198-00755.pd.

[2] Methocel molecular weight viscosity relationship. http://dowwolff.custhelp.com/app/answers/detail/a_id/1316.

[3] Segmental and chain dynamics in amorphous polymers. http://comse.chemeng.ntua.gr/segmdyn_polpage.htm.

[4] Taylan Altan, Gracious Ngaile, and Gangshu Shen. *Cold and hot forging: fundamentals and applications*, volume 1. ASM international, 2005.

[5] Ali S Argon. *The physics of deformation and fracture of polymers*. Cambridge University Press, 2013.

[6] B Avitzur. Metal forming: processes and analysis, 1968.

[7] Craig R Bartels, Buckley Crist, and William W Graessley. Self-diffusion coefficient in melts of linear polymers: chain length and temperature dependence for hydrogenated polybutadiene. *Macromolecules*, 17(12):2702–2708, 1984.

[8] M Doxastakis, DN Theodorou, G Fytas, F Kremer, R Faller, F Müller-Plathe, and N Hadjichristidis. Chain and local dynamics of polyisoprene as probed by experiments and computer simulations. *The Journal of chemical physics*, 119(13):6883–6894, 2003.

[9] Gerald Fleischer. Temperature dependence of self diffusion of polystyrene and polyethylene in the melt an interpretation in terms of the free volume theory. *Polymer Bulletin*, 11(1):75–80, 1984.

[10] Gerald Fleischer and Matthias Appel. Chain length and temperature dependence of the self-diffusion of polyisoprene and polybutadiene in the melt. *Macromolecules*, 28(21):7281–7283, 1995.

[11] Morton E Gurtin, Eliot Fried, and Lallit Anand. *The mechanics and thermodynamics of continua*. Cambridge University Press, 2010.

[12] Kenneth Langstreth Johnson. *Contact mechanics*. Cambridge university press, 1987.

[13] William Johnson and Peter Bassindale Mellor. *Engineering plasticity*. Horwood, 1983.

[14] CM Keary. Characterization of methocel cellulose ethers by aqueous sec with multiple detectors. *Carbohydrate polymers*, 45(3):293–303, 2001.

[15] Kyung Suk Kim and N Aravas. Elastoplastic analysis of the peel test. *International Journal of Solids and Structures*, 24(4):417–435, 1988.

[16] R Kimmich, W Unrath, G Schnur, and E Rommel. Nmr measurement of small self-diffusion coefficients in the fringe field of superconducting magnets. *Journal of Magnetic Resonance (1969)*, 91(1):136–140, 1991.

[17] Nikhil Padhye. *Sub-T_g, Solid-State, Plasticity-Induced Bonding of Polymeric Films and Continuous Forming*. PhD thesis, Massachusetts Institute of Technology, Cambridge, 2015.

[18] Nikhil Padhye, David M. Parks, Alexander H. Slocum, and Bernhardt L. Trout. Enhancing the performance of the t-peel test for thin and flexible adhered laminates. *Review of Scientific Instruments*, 87(8), 2016.

[19] DS Pearson, G Ver Strate, E Von Meerwall, and FC Schilling. Viscosity and self-diffusion coefficient of linear polyethylene. *Macromolecules*, 20(5):1133–1141, 1987.

[20] Mya Warren and Jörg Rottler. Microscopic view of accelerated dynamics in deformed polymer glasses. *Physical review letters*, 104(20):205501, 2010.
